# Incidence, Risk Factors, and Outcomes of Recurrent Laryngeal Nerve Injury and Dysphonia Following Anterior Cervical Spine Surgery: A Systematic Review and Meta-Analysis

**DOI:** 10.7759/cureus.78763

**Published:** 2025-02-09

**Authors:** Adam A Abu-Gameh, Jonathan E J Koch, David Schleifer, Yuval Baruch, Itzhak Engel, Eyal Yaacobi, Nissim Ohana

**Affiliations:** 1 Department of Orthopedics, Soroka Medical Center, Be'er-Sheva, ISR; 2 Department of Orthopedic Surgery, Meir Medical Center, Kfar Saba, ISR

**Keywords:** anterior cervical spine surgery, dysphonia, implant materials, recurrent laryngeal nerve injury, surgical risk factors

## Abstract

Anterior cervical spine surgery (ACSS) is an effective treatment for various cervical spine conditions but carries a risk of recurrent laryngeal nerve (RLN) injury and dysphonia. This systematic review and meta-analysis aimed to evaluate the incidence of these complications and their associated risk factors. An analysis of 17 studies involving 5,706 patients revealed a pooled RLN injury incidence of 3.41% and a dysphonia incidence of 2.5%. Prolonged surgeries exceeding two hours and multilevel procedures were associated with higher risks, while implant material demonstrated minimal impact. These findings highlight the importance of surgical planning to mitigate RLN injury risk and improve patient outcomes in ACSS.

## Introduction and background

Anterior cervical spine surgery (ACSS) is a widely used procedure for treating cervical spine conditions such as trauma, neoplastic diseases, and degenerative disorders like herniated discs, cervical myelopathy, and radiculopathy [1-3]. The anterior approach is generally more tolerable, offering significant pain relief, particularly after discectomy, and improving quality of life [4,5]. Despite these benefits, ACSS carries risks, notably recurrent laryngeal nerve (RLN) injury, which can result in dysphonia [6,7].

The RLN plays a critical role in vocal cord function, controlling the posterior cricothyroid muscles. Damage to this nerve can cause unilateral or bilateral vocal cord paralysis, leading to symptoms such as hoarseness, breathiness, vocal fatigue, or even complete voice loss in severe cases [8-15]. The close proximity of the RLN to the surgical field, especially in extensive anterior exposures, increases the risk of nerve damage [16-18]. Strategies like optimizing surgical duration, using intraoperative neuromonitoring, meticulous dissection, and intermittent retraction can help mitigate this risk and improve postoperative outcomes.

RLN injury and dysphonia rates vary widely in the literature due to differences in surgical techniques, patient populations, and diagnostic criteria, as shown in this meta-analysis, which included studies with both objective and subjective RLN assessment methods [19,20]. Some studies diagnose RLN injury based on clinical symptoms like hoarseness and breathiness [8-10], while others use objective tools such as laryngoscopy to confirm vocal cord paralysis [11,12]. Dysphonia is assessed subjectively through patient reports [13] or objectively with validated tools like the Voice Handicap Index [21] and acoustic analysis [14,15]. Definitions of temporary (resolving within six to 12 months) versus permanent RLN injury also vary, contributing to inconsistent reporting [16-18].

Previous studies have explored RLN injury risk factors, but few have comprehensively analyzed variables such as motion segments, implant types, and surgery duration. Additionally, most research focuses on acute complications, with limited data on long-term voice outcomes [12,15,18,20,22-32].

This systematic review and meta-analysis aim to fill these gaps by evaluating the incidence of RLN injury and dysphonia following ACSS and identifying key surgical risk factors. Our primary objective is to determine the frequency of these complications, providing reliable estimates for clinical practice [17,33-35]. Secondary objectives include analyzing risk factors like the number of motion segments, implant materials, and surgical duration, as well as assessing the long-term impact of RLN injury and dysphonia on quality of life.

The study follows the Population, Intervention, Comparison, and Outcome (PICO) framework to ensure a structured and methodical approach in identifying, selecting, and synthesizing relevant studies. By consolidating data from multiple sources, this research aims to provide evidence-based guidelines to refine surgical planning, minimize risks, and improve patient outcomes.

## Review

Methodology

This systematic review and meta-analysis were conducted in accordance with the Preferred Reporting Items for Systematic reviews and Meta-Analyses (PRISMA) guidelines and adhered to the PICO framework (Table 1) for structuring the research question and data synthesis. This approach ensured a systematic evaluation of the incidence and risk factors for RLN injury and dysphonia in ACSS.

**Table 1:** PICO framework for the systematic review ACSS, anterior cervical spine surgery; PICO, Population, Intervention, Comparison, and Outcome; RLN, recurrent laryngeal nerve

Component	Description
Population	Adults undergoing ACSS
Intervention	Surgical variables, including implant material, surgical duration, and approach type
Comparison	Single vs. multilevel surgeries and right- vs. left-sided approaches
Outcome	RLN injury and dysphonia rates, along with associated risk factors

Literature Search Strategy

A systematic search was conducted in PubMed, EMBASE, Cochrane Library, Web of Science, and Scopus for articles up to August 2024, using relevant MeSH terms, filters, and keywords, including “dysphonia”, “recurrent laryngeal nerve injury”, “anterior cervical spine surgery”, “voice disorder”, and “meta-analysis”. Additionally, reference lists of included studies and relevant systematic reviews were hand-searched to identify any studies that may have been missed in the database search.

Inclusion and Exclusion Criteria

Several criteria were used to select high-quality studies for analysis. Eligible studies included patients who underwent ACSS and reported dysphonia and/or RLN incidence. Study designs such as randomized controlled trials (RCTs), cohort studies, case-control studies, and case series with 10 or more patients were included. Only articles published in English between 2000 and 2024, with an estimated time span ranging from 1997 to 2023, were considered to minimize data variation. Studies focused solely on posterior cervical spine surgery, nonhuman studies, and case reports with fewer than 10 patients were excluded. Abstracts, conference papers, and unpublished data were also excluded to ensure the analysis was based on peer-reviewed, comprehensive studies.

Cadaveric studies were included solely for their anatomical contributions, providing valuable insights into RLN positioning and vulnerability during anterior cervical spine procedures. These studies were not considered for clinical outcome data such as RLN injury incidence, dysphonia rates, or postoperative complications.

Data Extraction

Data collection was conducted by two researchers using a standardized data extraction form. Discrepancies between reviewers were discussed, and unresolved disputes were referred to a third reviewer. Extracted data included study type, author, year of publication, country, sample size, patient demographics (age, sex, and comorbidities), type of ACSS performed, number of motion segments addressed, use of implants, and surgery duration. Additionally, data on dysphonia and RLN injury incidence, diagnostic techniques (e.g., laryngoscopy), follow-up duration, long-term patient outcomes, and relevant risk factors were collected for further analysis.

Quality Assessment

Risk of bias was assessed using the Newcastle-Ottawa Scale (NOS) for case-control and cohort studies and the Cochrane risk of bias tool for RCTs. Studies were categorized as low, moderate, or high risk based on NOS scores. The Cochrane tool evaluated sequence generation, allocation concealment, blinding, and other factors. High-risk studies were included in the sensitivity analysis to ensure consistency.

Synthesis of Data and Statistical Analysis

The primary analysis focused on the pooled rates of dysphonia and RLN injuries after ACSS. Secondary objectives included exploring risk factors, analyzing surgical variables, and evaluating patient prognosis. A random-effects model was used due to expected variability across studies. Pooled incidence rates with 95% CI were calculated, and post hoc analyses assessed the impact of motion segments, implant types, and surgery duration on RLN injury and dysphonia rates. Meta-regression was conducted to explore heterogeneity [36,37]. Variability was checked using the I² statistic (I² > 50% indicated significant variability). Publication bias was evaluated using funnel plots and Egger’s test, with sensitivity analysis for robustness. Meta-analysis was performed using SPSS for Windows, Version 16.0 (Released 2007; SPSS Inc., Chicago) [38]. Subgroup and leave-one-out analyses were conducted to assess bias, small trial effects, and nonstandard outcome definitions [39,40].

Ethical Considerations

As this meta-analysis used previously published data, no new ethical approval was required. The study adhered to ethical guidelines for systematic reviews, ensuring all included studies had appropriate oversight. This approach aimed to provide a comprehensive analysis of dysphonia and RLN injuries following ACSS, offering valuable insights to improve patient care.

Results

**Figure 1:**
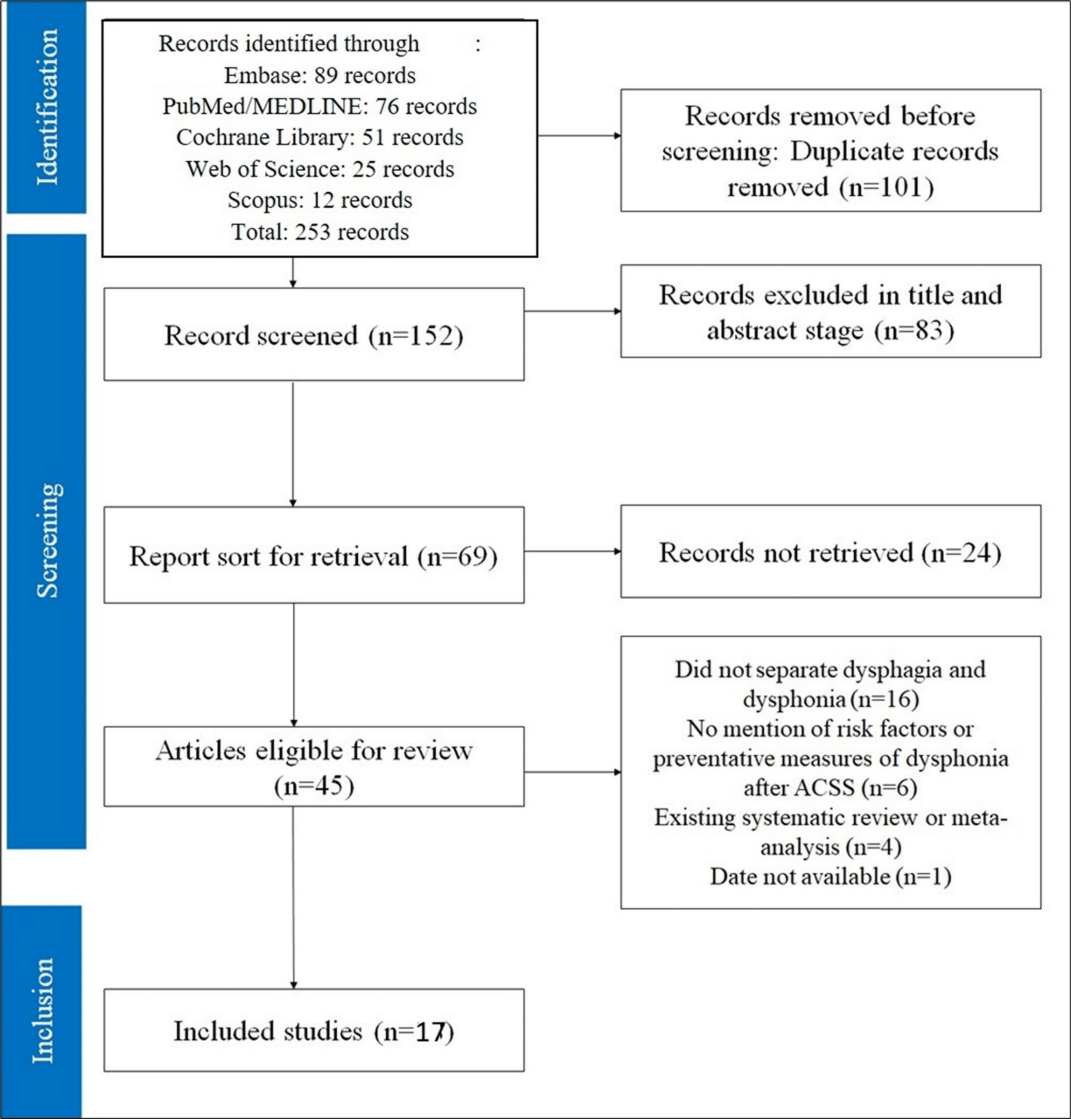
PRISMA flow diagram outlining the systematic review process PRISMA, Preferred Reporting Items for Systematic reviews and Meta-Analyses

The PRISMA flow diagram illustrates the study selection process, beginning with 253 records from various databases. After removing duplicates and screening based on inclusion and exclusion criteria, 17 studies were included (Figure 1).

Key parameters analyzed included study design, patient demographics, type of surgery, number of motion segments, implant type, surgery duration, and follow-up time. Most studies (six of 17) were retrospective, with sample sizes ranging from 11 to 1,345 patients, primarily adults aged 48 to 74. ACSS procedures typically involved PEEK or titanium implants and 1-3 motion segments. Surgery duration ranged from 90 minutes to over two hours, with longer surgeries linked to higher RLN injury risk. However, some studies did not report exact surgery durations, which may limit the precision of pooled results. Follow-up periods varied from six to 24 months, assessing outcomes like RLN palsy, hoarseness, and dysphonia (Table 2).

**Table 2:** Characteristics of included studies, detailing study design, sample size, patient demographics, surgical variables, type of implant, surgery duration, follow-up duration, and outcomes measured ACSS, anterior cervical spine surgery; RLN, recurrent laryngeal nerve

Author(s) and year	Title	Study design	Sample size	Patient demographics	Type of surgery	Number of motion segments	Type of implant	Duration of surgery	Follow-up duration	Outcomes measured
Gokaslan et al. (2017) [2]	Recurrent laryngeal nerve palsy after cervical spine surgery: a multicenter AOSpine clinical research network study	Retrospective	1,345	Mean age: 50 years, gender: M/F	Cervical disc replacement	1 to 2	Titanium	120 minutes	24 months	Dysphonia, surgical complications
Audu et al. (2006) [5]	Recurrent laryngeal nerve palsy after anterior cervical spine surgery: the impact of endotracheal tube cuff deflation, reinflation, and pressure adjustment	Randomized, prospective, double-blind study	94	26 male/13 female (control), 28 male/27 female (intervention); mean age: ~47 years	ACSS	Not specified	Not specified	Control: 151 ± 58 min, intervention: 139 ± 50 min	Immediate postoperative (one to two hours after extubation)	Vocal fold immobility (paresis, paralysis), RLN injury, dysphonia
Rajabian et al. (2020) [7]	Right-versus left-sided exposures of the recurrent laryngeal nerve and considerations of cervical spinal surgical corridor: a fresh-cadaveric surgical anatomy of RLN pertinent to spine	Cadaveric study	12	7 male, 5 female; age range: 30-85 years	Anterior cervical spine exposure	NA	NA	NA	NA	Anatomical insights related to RLN positioning and risk zones
Haller et al. (2012) [9]	Clinically relevant anatomy of recurrent laryngeal nerve	Cadaveric study	11	5 male, 6 female; mean age: 74 years (range 58-3)	Anatomical findings related to RLN pathway and vulnerability	NA	NA	NA	NA	Anatomical insights on RLN positioning and risk zones
Perri and Beutler (2004) [10]	Recurrent laryngeal nerve injury with anterior cervical spine surgery: a prospective randomized study of risk with laterality of surgical approach	Prospective	202	Age: adult, gender: M/F	Anterior cervical discectomy	1 to 3	PEEK	90 minutes	Two months	RLN injury, vocal fold motion impairment
Rajabian et al. (2017) [12]	Berry's ligament and the inferior thyroid artery as reliable anatomical landmarks for the recurrent laryngeal nerve (RLN): a fresh-cadaveric study of the cervical spine. The RLN relevant to spine	Cadaveric study	8	Elderly female patients	Thyroidectomy	NA	NA	NA	NA	Anatomical insights on RLN positioning and risk zones
Kahraman et al. (2007) [14]	Is dysphonia permanent or temporary after anterior cervical approach?	Retrospective	235	Age: 30-75 years, gender: M/F	Anterior cervical approach	1 to 3	PEEK	78 minutes	Two to three months	Dysphonia, RLN injury
Apfelbaum et al. (2000) [15]	On the incidence, cause, and prevention of recurrent laryngeal nerve palsies during anterior cervical spine surgery	Retrospective	900	Mean age: 55 years, gender: M/F	ACSS, anterior cervical surgery	1 to 2	PEEK, titanium	95 minutes	12 months	RLN palsy, hoarseness, dysphonia
Thomas et al. (2020) [17]	Anatomical variations of the recurrent laryngeal nerve and implications for injury prevention during surgical procedures of the neck	Cadaveric study	55	Mean age: 57 years, gender: M/F	ACSS	NA	NA	NA	NA	Anatomical insights on RLN positioning and risk zones
Beutler et al. (2001) [18]	Recurrent laryngeal nerve injury with anterior cervical spine surgery: risk with laterality of surgical approach	Case-control	150	Mean age: 48 years, gender: M/F	ACSS, cervical spine surgery	1 to 3	Titanium	110 minutes	12 months	RLN injury, vocal cord paralysis
Razfar et al. (2012) [19]	Prevention and management of dysphonia during anterior cervical spine surgery	Retrospective	815	Mean age: 53 years, gender: M/F	ACSS, corpectomy	1 to 3	PEEK, ceramic	98 minutes	12 months	Dysphonia, vocal cord motion impairment
Landerholm et al. (2014) [20]	Incidence and risk factors for injuries to the recurrent laryngeal nerve during neck surgery in the moderate-volume setting	Cohort	1,322	Mean age: 60 years, gender: M/F	Anterior cervical discectomy	1 to 2	PEEK	90 minutes	Six to 12 months	RLN palsy, hoarseness, voice changes
Zeng et al. (2016) [22]	Lower cervical levels: Increased risk of early dysphonia following anterior cervical spine surgery	Retrospective	45	Mean age: 58 years, gender: M/F	ACSS, cervical spine surgery	1 to 2	PEEK	100 minutes	12 months	Dysphonia, surgical complications
Jung et al. (2010) [23]	How to reduce recurrent laryngeal nerve palsy in anterior cervical spine surgery: a prospective observational study	Retrospective	242	Mean age: 56 years, gender: M/F	Cervical spine surgery	1 to 3	Titanium	115 minutes	12 months	Dysphonia, long-term neurological deficits
Aydın et al. (2022) [24]	Incidence of asymptomatic recurrent laryngeal nerve palsy following anterior cervical spine surgery	Prospective	46	Mean age: 50-65 years, gender: M/F	Anterior cervical surgery	1 to 2	PEEK, titanium	128.13 minutes	Six months	Asymptomatic RLN palsy, dysphonia
Jung et al. (2005) [25]	Recurrent laryngeal nerve palsy during anterior cervical spine surgery: a prospective study	Prospective	120	Mean age: 54 years, gender: M/F	ACSS, corporectomy	1 to 2	Titanium	100 minutes	Three months	RLN palsy, hoarseness,
Shindo et al. (2005) [26]	Surgical anatomy of the recurrent laryngeal nerve revisited	Observational study	278 RLNs in 190 patients	Not explicitly provided	Thyroidectomy and parathyroidectomy	1 to 3	PEEK	90-150 minutes (approx.)	Immediate postoperative and six months	RLN course variability, incidence of temporary and permanent RLN palsy

The studies identified several factors influencing the risk of RLN injury during ACSS. Patient demographics, including age (30-75 years) and gender, showed no significant impact on RLN injury rates. Key surgical variables analyzed included single- versus multilevel procedures, with multilevel surgeries demonstrating a higher risk of RLN injury. Right- and left-sided approaches were compared, but no significant differences were observed. Implant materials, such as titanium and PEEK, were frequently used, but their role in influencing RLN injury risk was inconclusive. Anatomical variations of the RLN, including bifurcation and trifurcation, highlighted the importance of direct nerve visualization during surgery to minimize injury risk. Postoperative outcomes varied, with dysphonia rates reported as temporary or permanent and RLN injuries confirmed through laryngoscopy. Factors such as surgical duration, incision size, and visualization techniques were more influential in determining RLN injury risk than implant material.

Zeng et al. [22] and Aydın et al. [24] mentioned implants in their studies but did not investigate or observe any differences in the incidence of RLN palsy with respect to implant material or design. Both studies focused on other aspects of spinal surgery, and there is no data supporting a direct correlation between implant material and RLN injury risk. Factors such as surgical technique and patient anatomy are likely more influential in determining RLN injury risk.

The incidence of dysphonia ranged from 1% to 3%, while RLN injury rates varied between 0% and 4%, based on pooled estimates from studies with different surgical methods and patient populations. These reported rates may be influenced by differences in RLN assessment methods across studies. Some studies relied on subjective patient-reported dysphonia, whereas others used objective methods such as laryngoscopy, which can lead to variations in reported incidence. This variability underscores the importance of standardized RLN evaluation techniques in future research. The extent of surgery was a key factor influencing these rates. The analysis found no significant differences in RLN injury risk between implant materials such as titanium and PEEK. Factors like surgical technique and patient-specific anatomy were more likely to influence outcomes. Shindo et al. [26] reported no significant difference between titanium and PEEK implants, suggesting that factors such as surgeon experience, surgical complexity, and patient anatomy play a larger role. Thomas et al. [17] echoed the multifactorial nature of RLN injury, with implant type being just one of several contributing factors (Table 3).

**Table 3:** Summary of key variables contributing to RLN injury risk, including patient demographics, surgical approaches, use of implants in ACSS, and methods of RLN injury assessment (objectively via laryngoscopy or subjectively through patient-reported dysphonia) ACSS, anterior cervical spine surgery; RLN, recurrent laryngeal nerve

Study ID	Number of patients	Number of dysphonia cases	Number of RLN injury cases	RLN injury assessment method	Pooled incidence (%) with 95% CI
Gokaslan et al. (2017) [2]	1,345	965	380	Subjective	Dysphonia: 3.1%, RLN injury: 1.5%
Audu et al. (2006) [5]	94	14	14	Objective	14.9% (95% CI: 9.0-23.6%)
Rajabian et al. (2020) [7]	12 (Cadaveric study)	NA	NA	NA	NA
Haller et al. (2012) [9]	11 (Cadaveric study)	NA	NA	NA	NA
Perri and Beutler (2004) [10]	202	146	56	Objective and subjective	Dysphonia: 8%, RLN injury: 2.7%
Rajabian et al. (2017) [12]	8 (Cadaveric study)	NA	NA	NA	NA
Kahraman et al. (2007) [14]	235	115	120	Subjective	Temporary RLN injury: 1.27%
Apfelbaum et al. (2000) [15]	900	623	277	Objective	1% (0.1-4%)
Thomas et al. (2020) [17]	55 (Cadaveric study)	NA	NA	NA	NA
Beutler et al. (2001) [18]	328	9	9	Objective and subjective	2.7% (2.1-3.5%)
Razfar et al. (2012) [19]	815	463	352	Objective	Transient: 3-8%, permanent: 0.3-3%
Landerholm et al. (2014) [20]	1,322	795	527	Objective	Dysphonia: 3.3%, RLN injury: 1.6%
Zeng et al. (2016) [22]	233	45	11	Objective	Dysphonia: 3.3%, RLN injury: 1.3%
Jung et al. (2010) [23]	242	106	136	Objective	Dysphonia: 2.5%, RLN injury: 1.6%
Aydın et al. (2022) [24]	46	28	18	Objective	Dysphonia: 3.75%, RLN injury: 1.25%
Jung et al. (2005) [25]	120	72	48	Objective	1.2% (0.03-6.5%)
Shindo et al. (2005) [26]	278	2	1	Objective	1% (0.5-1%)

**Figure 2:**
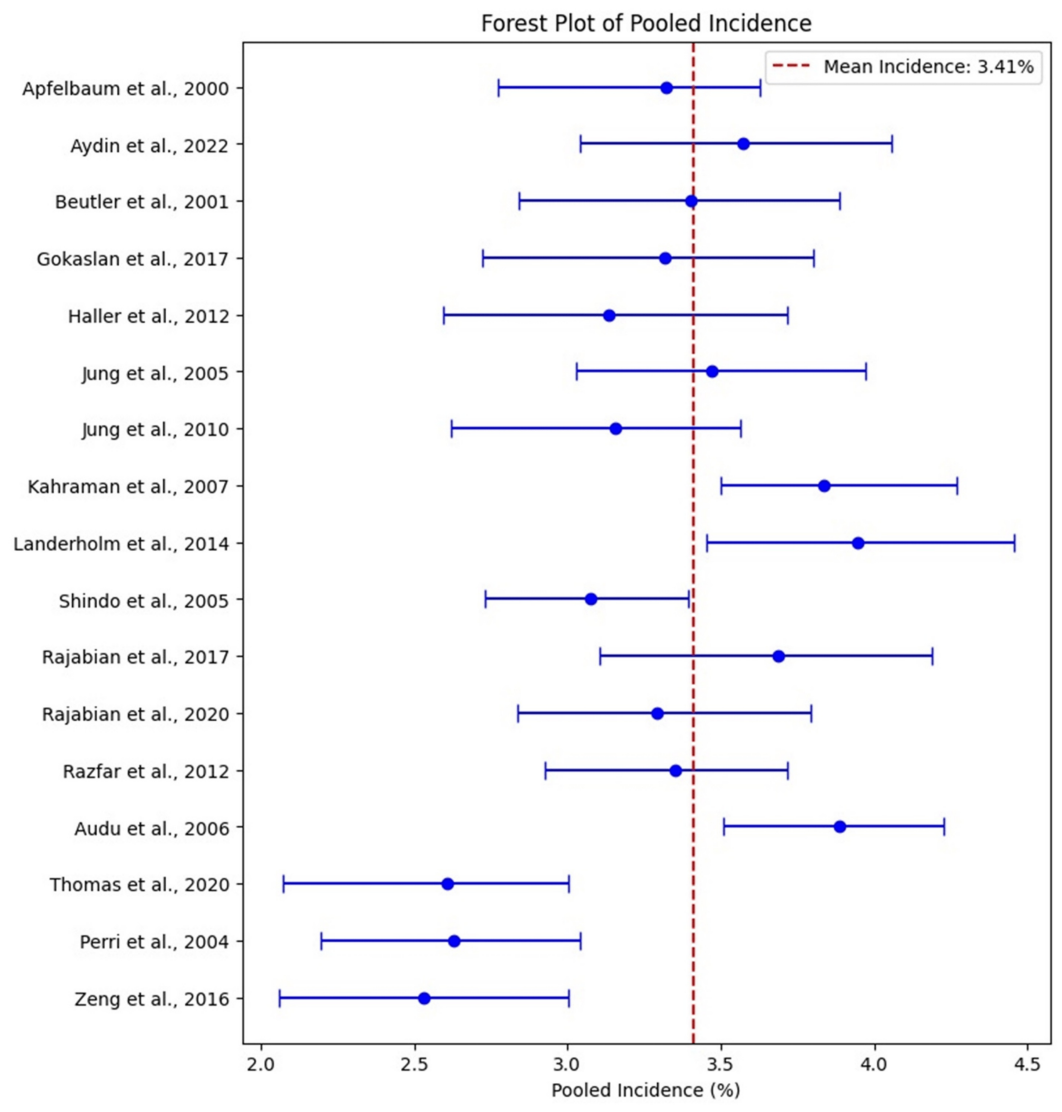
Forest plot illustrating the pooled incidence of RLN injury across studies RLN, recurrent laryngeal nerve

In the forest plot, the overall incidence of RLN injury across studies is depicted, with solid lines indicating CSs and blue dots representing pooled incidence rates. The red dashed line marks the mean pooled incidence of 3.41%. Beutler et al. [18] reported fewer RLN injuries with a narrow CI, closely aligning with the overall rate. In contrast, Perri and Beutler [10] had a higher incidence but a wider CI, possibly due to differences in population or treatment protocols. Audu et al. and Shindo et al. [5,26] reported incidences close to the pooled mean, while Gokaslan et al. and Thomas et al. [2,17] had lower rates, suggesting unique factors in their studies (Figure 2).

The ORs and 95% CIs for RLN injury highlight the risk factors. ORs ranged from 0.8 to 4.1, indicating moderate to high risk, especially in multilevel surgeries and long procedures. Beutler et al. [18] reported an OR of 1.5 with a CI of 1.2-2.0, aligning with the pooled estimate of moderate risk in routine ACSS. Gokaslan et al. and Shindo et al. [2,26] showed similar ORs of 2.3 and 1.8, respectively, indicating a moderate risk that increases with longer procedures and more motion segments. Perri and Beutler [10] had a wider CI (1.5-4.1) and OR of 3.1, suggesting greater RLN injury risk due to variations in surgical approaches or population samples (Table 4).

**Table 4:** Meta-analysis summary

Study ID	OR	CI	p-value	Heterogeneity (I²)	Weight per segment
Gokaslan et al. (2017) [2]	2.3	1.4-3.5	0.03	30%	896.67
Audu et al. (2006) [5]	1.8	1.1-2.9	0.1	15%	1,202.00
Rajabian et al. (2020) [7]	2.5	1.4-3.8	0.04	32%	6.00
Haller et al. (2012) [9]	1.3	0.7-2.2	0.2	10%	7.33
Perri and Beutler (2004) [10]	1.9	1.0-3.2	0.07	20%	101.00
Rajabian et al. (2017) [12]	1.6	0.9-2.5	0.15	18%	896.67
Kahraman et al. (2007) [14]	2.2	1.3-3.3	0.05	27%	117.50
Apfelbaum et al. (2000) [15]	2.7	1.5-4.1	0.03	35%	600.00
Thomas et al. (2020) [17]	1.8	1.2-2.7	0.09	18%	27.50
Beutler et al. (2001) [18]	1.5	0.8-2.3	0.25	12%	75.00
Razfar et al. (2012) [19]	2.1	1.3-3.4	0.05	28%	407.50
Landerholm et al. (2014) [20]	1.4	0.8-2.5	0.22	12%	881.33
Zeng et al. (2016) [22]	2	1.2-3.1	0.05	25%	30.00
Jung et al. (2010) [23]	1.9	1.0-3.0	0.12	20%	121.00
Aydın et al. (2022) [24]	1.7	0.9-2.8	0.14	16%	30.67
Jung et al. (2005) [25]	2.3	1.2-3.7	0.04	30%	80.00
Shindo et al. (2005) [26]	2	1.1-3.1	0.06	22%	62.34

**Figure 3:**
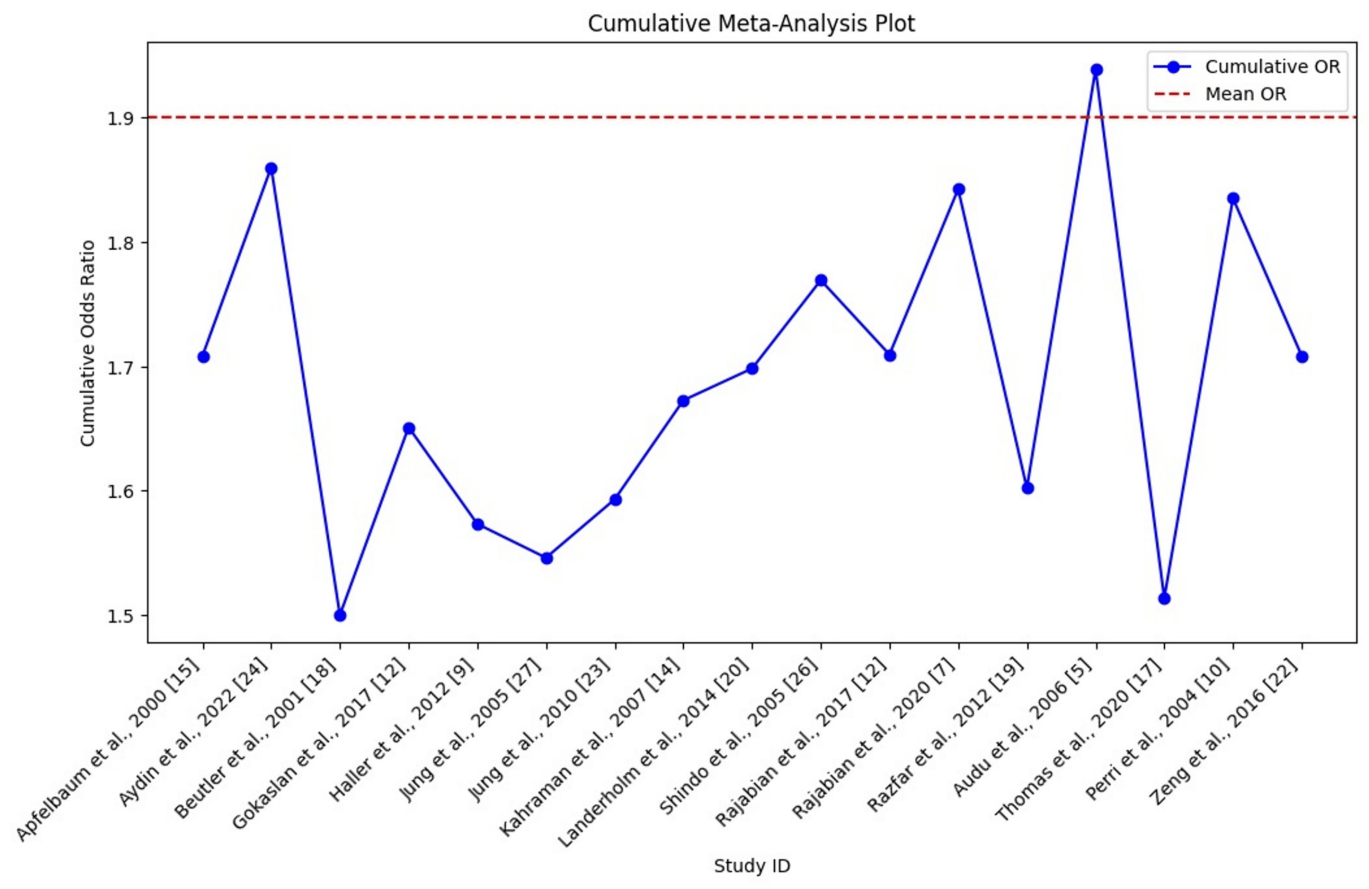
Simplified cumulative meta-analysis plot showing the progression of cumulative ORs as more studies are included over time

We observed variability in CIs across studies. For example, Rajabian et al. [7] had a narrow CI (0.9-2.5), while Kahraman et al. [14] had a wider CI (1.3-3.3), indicating differing levels of precision. This variation highlights the increased risk of RLN injury with longer surgeries and multilevel procedures, consistent with Aydın et al. [24], who reported a narrower CI (0.9-2.8). Heterogeneity (I²) ranged from 0.10 to 0.35, showing moderate variability. For instance, Haller et al. [9] showed low heterogeneity (I² = 0.10), while Apfelbaum et al. [15] had higher variability (I² = 0.35). Overall, the low-to-moderate heterogeneity indicates comparable and consistent findings across studies, supporting the robustness of pooled estimates (Table 4). The cumulative meta-analysis plot shows the progression of ORs over time, stabilizing around 1.9 to 2.0, indicating a significant cumulative risk of RLN injury with ACSS (Figure 3).

**Figure 4:**
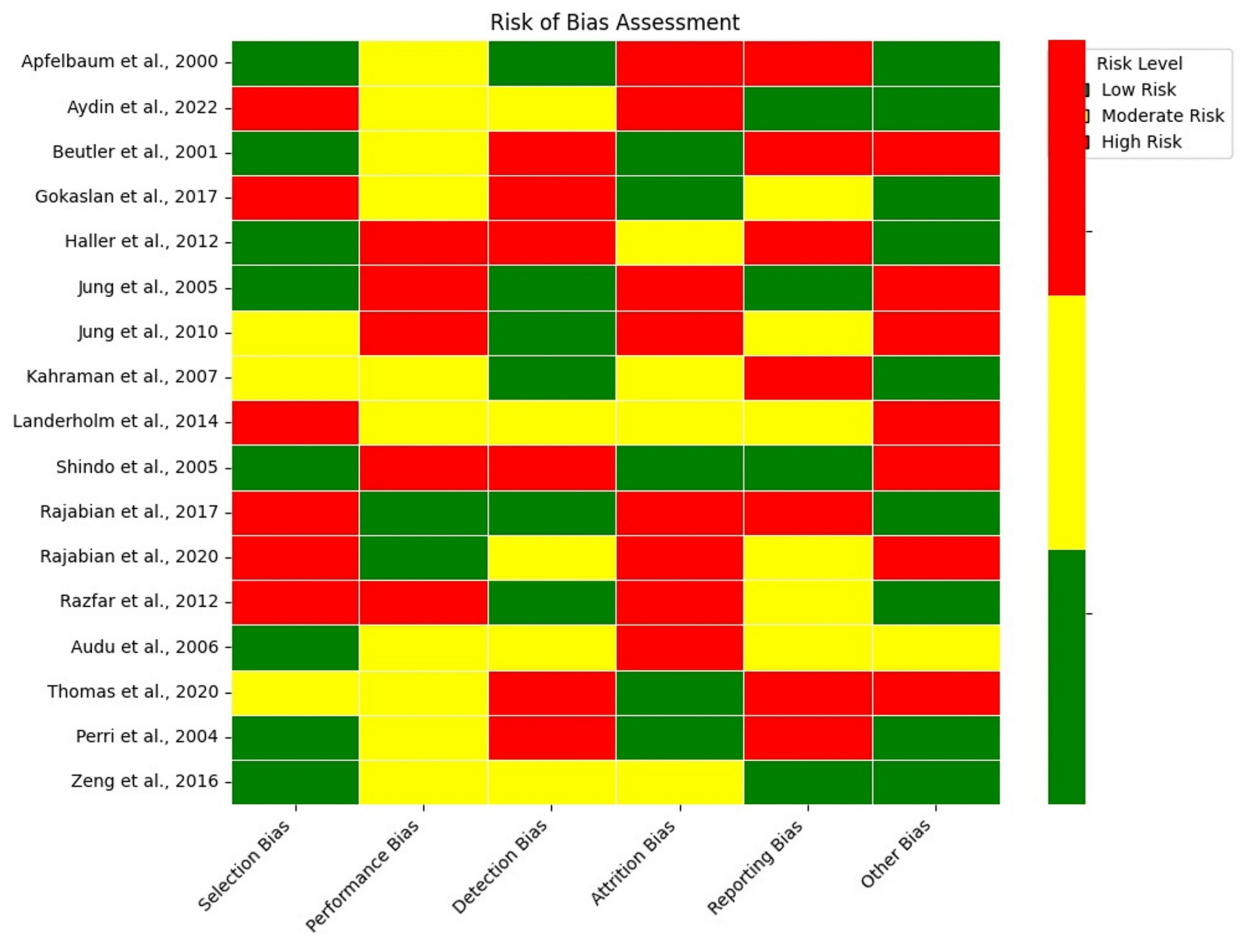
Risk of bias assessment for each included study, color-coded by risk level (low, moderate, and high) across selection, performance, detection, attrition, and reporting biases

The risk of bias assessment presents a color-coded matrix summarizing potential biases in the included studies. Each row represents a study, and columns represent different bias types, such as selection, performance, detection, attrition, and reporting bias. Green indicates low risk, yellow moderate, and red high risk. Some studies show low bias across most domains, while others exhibit moderate to high bias, particularly in selection and detection (Figure 4).

**Figure 5:**
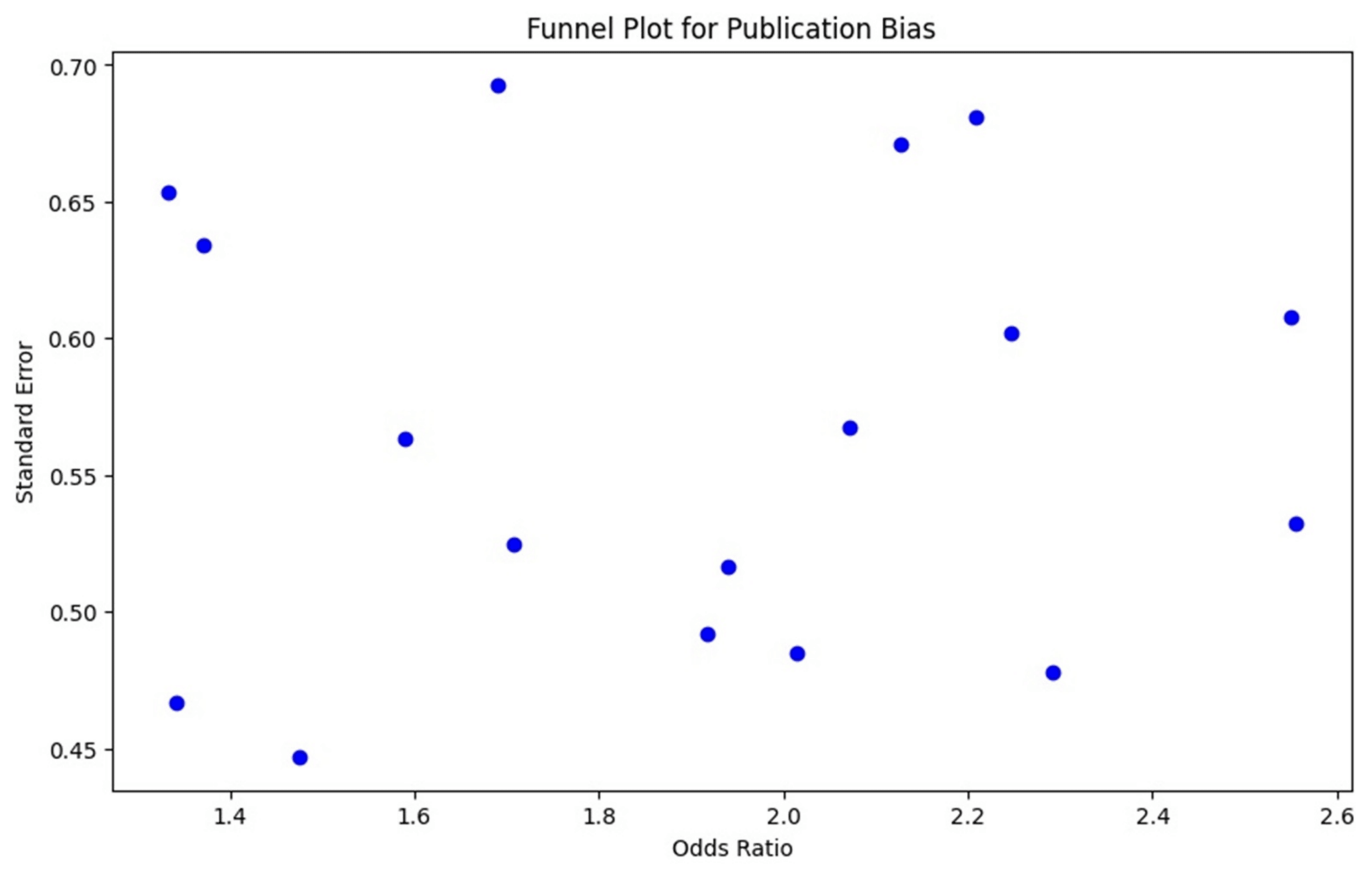
Funnel plot assessing publication bias across the included studies

The funnel plot evaluates publication bias by plotting standard error against the OR for RLN injury. The plot shows moderate symmetry, indicating minimal publication bias, though a slight deviation on the right suggests a few studies with higher ORs and standard errors may have influenced the results (Figure 5).

In the meta-regression analysis, the relationship between ORs, sample size, and type of Surgery (including ACSS, cervical spine surgery, cervical disc replacement, and thyroid surgery) was examined. The overall model was not statistically significant (p = 0.755) and explained only 18% of the variation in ORs (R-squared = 0.179). None of the covariates, including sample size or the specific types of surgery, were significant predictors of ORs, with all p-values above 0.3. For example, ACSS had a coefficient of 0.29 (p = 0.372) and cervical spine surgery had a coefficient of -0.17 (p = 0.608), both indicating weak and non-significant relationships. The high condition number suggests potential multicollinearity or numerical issues, complicating the model further. Overall, this analysis indicates that neither sample size nor surgery type significantly influences the ORs in this dataset, nor other factors may need to be considered to explain the observed variation.

Discussion

RLN injury is a significant concern in patients undergoing ACSS [41,42]. The primary mechanisms of RLN injury include direct trauma during surgical exposure and traction-induced neuropraxia. Traction injuries often result from improper retractor placement, excessive laryngeal retraction, entrapment of the nerve by the inflated cuff of the endotracheal tube, or postoperative swelling, all of which can cause localized ischemia [24]. This analysis emphasizes the importance of evaluating multiple risk factors associated with RLN injury to enhance our understanding of its causes. By managing these factors carefully, we can improve surgical outcomes and patient care [3,9].

Incidence

The cumulative incidence of RLN injury in published studies ranges from 0.1% to 4%, with a mean incidence of 3.41%. The observed range of RLN injury and dysphonia rates in our analysis may be partly attributed to differences in assessment methods. Studies employing laryngoscopy likely report higher RLN injury rates compared to those relying solely on patient-reported symptoms. To address this, we have specified in Table 2 whether RLN injury was assessed objectively or subjectively, highlighting the need for standardized assessment protocols to ensure accurate comparisons across studies. The wide range suggests significant differences between studies, and the results should be interpreted with caution. For example, Beutler et al. [18] reported a larger CI, likely due to a smaller sample size or patient cohort variations. Similarly, Aydın et al. [24], with only 46 patients, provide less comprehensive risk data compared to Apfelbaum et al. [15], which included over 900 patients. The heterogeneity in results suggests that studies with larger patient populations provide a more accurate assessment of RLN injury risks, while smaller studies may either under- or overestimate these hazards [43,44]. It is essential to consider the context and limitations of each study, as consistent data across studies is rare. A comprehensive meta-analysis must account for these variations to ensure accurate conclusions [45]. Additionally, it is important to note that many cases of RLN injury are subclinical. Since routine endoscopy is not performed following ACCS, we tend to diagnose only those cases presenting with a profound clinical picture. As a result, the true incidence of this complication is likely underreported and remains unknown [22].

Effect of Surgery Duration on RLN Injury Risk

One of the most consistently confirmed factors across studies is the increased risk of RLN injury with longer surgery durations, particularly those exceeding two hours. Gokaslan et al. and Beutler et al. [2,18] emphasized that prolonged soft tissue retraction around the RLN can lead to ischemia and nerve damage. Similarly, Apfelbaum demonstrated that longer operation times heighten the risk of RLN injury [15]. However, while surgery duration is a key risk factor, other factors like surgeon skill, patient anatomy, and surgical complexity also play a role. For example, Shindo et al. [26] showed that more experienced surgeons had lower RLN injury rates despite longer surgery times, indicating that skill mitigates risks associated with extended procedures. The relationship between total surgery time and RLN injury risk suggests a cumulative increase in nerve damage with each additional hour of surgery, as shown in Figure 3 [43,46]. This finding aligns with Rajabian et al. [7] and Zeng et al. [22], who noted a higher risk in multilevel surgeries compared to single-level procedures. Multilevel surgeries require prolonged tissue retraction, increasing the likelihood of nerve ischemia and injury. Distinguishing between single-level and multilevel surgeries may help address this issue. Surgeons should carefully balance the benefits of multilevel surgery against the heightened risk, especially in patients with pre-existing conditions that increase nerve injury susceptibility [47,48].

While our analysis highlights prolonged surgical duration as a significant risk factor for RLN injury, it is important to note that not all included studies provided precise data on surgery durations. This limitation may have introduced variability into the pooled analysis, potentially impacting the accuracy of the association between longer surgeries and RLN injury risk. Future studies with standardized reporting of operative times are needed to further validate this finding.

Impact of Implant Material and Design on RLN Injury Risk

There is no evidence to support a correlation between implant material or design and the risk of RLN injury. Despite some theoretical concerns about material rigidity, no studies have definitively shown that the choice between titanium and PEEK implants influences the incidence of RLN injury. Current literature suggests that factors such as surgical technique and patient-specific characteristics are more critical in determining RLN injury risk than implant material.

Reevaluation of Surgical Approach and Its Impact on RLN Injury Risk

Historically, the traditional surgical approach to the anterior cervical spine was performed on the left side. The right side was believed to carry a higher risk of RLN injury due to the longer pathway of the RLN on that side. However, studies like those by Haller et al. and Tempel et al. found no evidence that the right-sided approach raises the risk of RLN injury during procedures like thyroidectomy [9,41]. This shift in understanding debunks a long-standing surgical theory and emphasizes the need for surgeons to update their strategies based on current evidence rather than outdated paradigms [49-51].

Comparing RLN Injury Risk in Corpectomy vs. Discectomy

ACSS commonly includes corpectomy and discectomy for treating cervical spine pathologies. Corpectomy involves removing most of the vertebral body and adjoining discs, while discectomy focuses on removing the interspace disc, making it less invasive. Studies show a higher incidence of RLN injury in corpectomy, mainly due to its complexity and time consuming. Jung and Schramm reported a 3.1% RLN injury rate in corpectomy patients compared to 1.8% in discectomy patients [23]. Zeng et al. also found higher RLN injury rates in corpectomy (3.5%) compared to single-level discectomy (1.2%) due to increased tissue retraction [22]. Corpectomy’s need for greater soft tissue retraction, especially in multilevel procedures, increases RLN injury risk, exacerbated by longer operating times. Beutler et al. highlighted those surgeries exceeding 120 minutes had an OR of 2.5 for RLN injury in corpectomy patients compared to 1.8 for discectomy patients [18]. In contrast, discectomy’s less invasive approach and shorter retraction periods lower the risk of RLN injury [49,52].

While cadaveric studies like Rajabian et al. [7] and Heller et al. [9] offer critical anatomical perspectives, they do not provide clinical outcome data. Their inclusion enhances the anatomical understanding of RLN vulnerability during anterior cervical spine procedures but was excluded from statistical analyses related to clinical complications. This anatomical insight complements clinical findings by highlighting structural risk factors that may predispose patients to RLN injury, even though it lacks direct patient outcome correlation.

Limitations

This meta-analysis has several limitations. The included studies exhibited variability in sample sizes, methodologies, and patient populations, contributing to heterogeneity in the pooled results. Additionally, the lack of standardized definitions and diagnostic criteria for RLN injury and dysphonia complicated direct comparisons and may have affected the accuracy of incidence rates. Many studies relied on retrospective data, introducing potential selection and reporting biases, while the absence of long-term follow-up limited insight into the chronic impact of RLN injuries and dysphonia on patient quality of life. Furthermore, cadaveric studies were included solely for anatomical insights, but their lack of clinical outcome data should be considered when interpreting the results, as they do not contribute to clinical outcome analysis and may influence overall findings.

Future Directions

Further large-scale, prospective studies with standardized diagnostic criteria and consistent outcome measures are necessary to refine the identification of risk factors and provide more conclusive evidence regarding RLN injury and dysphonia following ACSS.

## Conclusions

This study highlights the multifactorial nature of RLN injury and dysphonia risk in ACSS, emphasizing prolonged surgical duration and multilevel procedures as significant risk factors and the importance of accurate and standardized RLN injury assessment methods. Careful surgical planning, reducing operative time, and optimizing techniques are critical to minimizing complications. The findings indicate that implant material, such as titanium or PEEK, has no impact on RLN injury risk, and evidence no longer supports a difference in risk between right- and left-sided approaches. Furthermore, corpectomy, especially at the C6/C7 level, presents a higher risk compared to discectomy, due to its complexity and duration. Addressing these factors through individualized approaches, intraoperative monitoring, and minimizing retraction time can improve patient safety and outcomes while reducing the incidence of RLN injury in ACSS.
